# Twelve Tips to Train Medical Students to Manage Their Uncertainty and to Provide Reassurance to Patients and Their Caregivers

**DOI:** 10.1007/s40670-025-02402-y

**Published:** 2025-05-08

**Authors:** Colin J. McMahon, Muirne Spooner, Dimitrios Papanagnou, Matthew Sibbald, Maryam Asoodar

**Affiliations:** 1 Department of Paediatric Cardiology, Children’s Health Ireland at Crumlin, Dublin, Ireland; 2 School of Medicine, University College Dublin, Belfield, Dublin 4, Ireland; 3 School of Health Professions Education (SHE), Maastricht University, Maastricht, Netherlands; 4 Department of Medicine, Royal College of Surgeons University of Medicine and Health Sciences, Dublin, Ireland; 5 Department of Internal Medicine, Jefferson College, Philadelphia, PA USA; 6 Department of Medicine, Faculty of Health Sciences, McMaster University, Hamilton, ON Canada

**Keywords:** C4/ID model, Comfort with uncertainty, Decision-making, Empathy, Paediatrics, Uncertainty, Tips, Training

## Abstract

**Background:**

Uncertainty is pervasive in clinical medicine and provides a major hurdle for decision-making. Our previous work has demonstrated the importance of providing parents of paediatric patients reassurance in the medical team’s plan of action, what we have termed “comfort with doctors’ reassurance.”

**Aims:**

We provide several practical and implementable tips to medical students in how they can learn to deal with their own uncertainty, recognize the subtle complexities involved and acknowledge the emotional stress that can accompany this process, develop a healthy long-term relationship with uncertainty, and ultimately embrace its potential in providing holistic care to their patients and their caregivers.

**Results:**

Twelve tips and actions in four main domains are recommended: (A) Understanding and Integrating Uncertainty into Medical Education, (B) Building Resilience and Empathy Through Self-Regulation and Reflection, (C) Enhancing Communication and Relationship-Building Skills, and (D) Clinical Skills for Reassurance and Decision-Making.

**Conclusions:**

Even though uncertainty in medical decision-making is pervasive and challenging for the medical trainee, there are several concrete strategies that can build comfort with uncertainty in trainees and reinforce the potential positive attributes of uncertainty in the holistic care of patients. Empathy and expressing compassion remain key skills in bridging caregiver discomfort with uncertainty and comfort with doctors’ reassurance.

## Introduction

Uncertainty is pervasive in clinical medicine and adds to the challenge in medical decision-making [1, 2]. Over the last decade, as an example, there has been a dramatic increase in referral of patients to paediatric cardiology clinics [3] for a multitude of reasons including lack of physician and parental comfort with uncertainty, parental anxiety and parental pressure on medical professionals to refer for further specialist evaluation. There is increasing recognition of the value on preparing clinicians and trainees in the health professions to manage their tolerance of ill-defined problems in clinical practice [4–8]. Promoting the capacity of trainees to deal with this uncertainty in clinical contexts may have tangible effects on patient care and how scarce resources are allocated [3]. This is particularly pertinent in the current climate of cost-conscious quality care, but more importantly, alleviating parental and patient anxiety surrounding their uncertainty.

Many investigators have focussed on the importance and management of uncertainty in clinical medicine [9, 10]. Several researchers have explored the associations between practitioner or trainee “uncertainty tolerance” with healthcare related outcomes [9, 11–13]. Strout et al. [11] correlated lower uncertainty tolerance and adverse outcomes, including reduced practitioner/trainee wellbeing poorer attitudes toward person-centered care [8, 11, 14–16]. As a result, being able to reflect on their uncertainty tolerance is now recognized as an important skill for medical trainees [8, 9, 17, 18].

Many of the terms which have evolved around uncertainty in themselves generate a degree of confusion: uncertainty tolerance, comfort with uncertainty, discomfort with uncertainty, uncertainty orientation, ambiguity tolerance and stress from uncertainty are all terms which have been applied to uncertainty by different authors. For medical practitioners, educators or trainees, these can become a bit overwhelming not to mention frustrating [9]. Hillen et al. attempted to dispel this confusion and to bring some standardization to uncertainty tolerance by clearly defining it as “the set of negative and positive psychological responses—cognitive, emotional and behavioural—provoked by the conscious awareness of ignorance about particular aspects of the world” [19].

Gerrity et al. and Hillen et al. further refined our understanding of being “tolerant” of uncertainty [7, 19]. Ilgen et al. discussed the importance of feeling stressed in managing uncertainty, as this effectively signals the need for support to the trainee [20]. Several authors have also identified specific moderators with impact on trainee reaction to their uncertainty [12, 21]. These researchers postulate that uncertainty tolerance can be adapted with increasing experience rather than being a fixed trait [9]. Stephens and Lazarus proposed adopting a more nuanced perception of uncertainty tolerance, evolving from a binary one to one which factors in whether a set of responses (i.e., emotional, behavioral, and cognitive) are maladaptive or merely adaptive when responding to uncertainty [9].

Previously our work has demonstrated the importance of providing parents of paediatric patients reassurance in the medical professionals plan of action, what we term “comfort with doctors reassurance” (CDR) [21]. Five themes were identified: the interplay between quality of information, uncertainty and decision-making, confidence in clinical assessment and first-hand patient evaluation, anxiety and fear experienced by medical professionals when dealing with complex and serious conditions, strategies employed by medical professionals in managing their own uncertainty and the impact of societal and parental expectations on medical decision-making. These are moderated by a number of factors, most significantly the child’s caregivers’ comfort with doctors reassurance (CDR) [21]. Medical practitioner comfort with uncertainty is inextricably intertwined with parental or caregiver CDR. Comfort with reassurance builds upon the concept of reassurance for medical patients by enhancing the patient’s experience and fostering trust in the healthcare provider. When medical students are comfortable in their ability to reassure, they communicate confidence and genuine concern, which can reduce patients’ anxiety and fears.

This work builds upon previous work but provides further tips to manage uncertainty where current gaps exist. Herein we present 12 tips for trainers to help students navigate uncertainty and provide CDR to caregivers of patients with benign conditions. Each tip includes two components: first, the theoretical underpinning of each tip and second, an action plan to implement concrete steps to train the healthcare professional trainee in managing their uncertainty.

## Pedagogical Tips to Equip Medical Trainees to Navigate Uncertainty in Healthcare

The goal of these tips is to create a supportive learning environment where medical students can develop the necessary knowledge, skills, and behaviors to manage uncertainty in their practice. Incorporating uncertainty into their practice of holistic care allows them to develop a more comprehensive understanding of their patients. By fostering thorough assessments, encouraging collaborative decision-making, promoting continuous learning, and enhancing empathy, uncertainty can lead to more effective and compassionate care. These skills are key for future healthcare professionals who aim to provide holistic and patient-centered care in an increasingly complex medical environment.

The uncertainty literature has exploded over the last few years. Several authors have undertaken scoping reviews [18], reported techniques to aid clinicians in managing their uncertainty [22], evaluated what tips promote learners to manage their uncertainty [9] and even integrated uncertainty training into formal undergraduate medical curricula [23].

Four overarching strategies are key to navigating uncertainty: (A) Understanding and Integrating Uncertainty into Medical Education, (B) Building Resilience and Empathy Through Self-Regulation and Reflection, (C) Enhancing Communication and Relationship-Building Skills, and (D) Clinical Skills for Reassurance and Decision-Making.

### Understanding and integrating uncertainty into medical education

These tips emphasize theoretical foundations and systemic strategies to introduce the concept of uncertainty in medical education. They explore how trainees can develop an awareness of the pervasiveness of uncertainty and build the cognitive and emotional tools to handle it. Together, they create a supportive structure where understanding uncertainty becomes a key educational goal.

• Tip 1: Exposing students to different types of uncertainty early in training

Medical students should be introduced to the concept of medical uncertainty and its natural history early in their training. According to Han, responses to uncertainty can be categorized into two main types: primary and secondary [24].

1. **Primary responses**: These are direct reactions from both clinicians and patients, which can include:
  A. **Risk aversion**: Reluctancy to engage in situations where the outcomes are uncertain.
  B. **Ambiguity aversion**: Avoiding scenarios where information is incomplete or unclear.
  C. **Complexity aversion**: Hesitancy to deal with multifaceted cases that require intricate decision-making.
2. **Secondary responses**: These involve compensatory strategies to manage the discomfort associated with uncertainty, including:
  A. **Cognitive responses**: Such as feelings of vulnerability and pessimism regarding risks and benefits.
  B. **Emotional responses**: Including fear, worry, and anxiety that can arise when faced with uncertain situations.
  C. **Behavioral responses**: Actions like decision avoidance or deferring to others when making clinical choices [24].

## Classification of strategies to manage uncertainty according to Han [24]

**Table:** 

Strategy	Focuses	Approaches	Examples
Ignorance-focused	Avoids or ignores uncertainty	Avoidance, over-simplification	Ignoring patient complexities
Uncertainty-focused	Acknowledges and embraces uncertainty	Exploration, adaptability	Discussing treatment uncertainties
Response-focused	Proactively manages challenges	Proactivity, skill development	Simulation training for critical care
Relationship-focused	Builds and maintains interpersonal connections	Collaboration, empathy	Team debriefing sessions

To aid in managing uncertainty, Patel et al. proposed the RAPS framework, which stands for Recognize, Acknowledge, Partner, and Seek support [25]. This framework is designed to help both students and clinicians navigate uncertainty more effectively.

Further, Rusnack et al. developed a virtual, immersive case study aimed at challenging medical students to better understand and address clinical uncertainty [26]. In this study, students utilized sensemaking frameworks to:

- Analyze clinical scenarios
- Identify sources of uncertainty (e.g., diagnostic, practical, or personal)
- Classify these uncertainties within the Cynefin framework, a decision-making support tool developed by Snowden and Boone (2007) [27]. The Cynefin framework categorizes issues into five distinct domains, each requiring different approaches for effective decision-making including clear, complex, complicated, chaotic, and aporetic (confused) domains, which provides a structured approach to understanding and responding to diverse challenges. This framework is particularly useful in healthcare.

This immersive approach enables students to recognize the nature of uncertainty, reflect on its implications for decision-making, and formulate tailored problem-solving strategies using available resources and communication tools.

### Action

Incorporate various strategies into the curriculum to help students manage uncertainty. These include collecting more information, embracing uncertainty e.g. through mindfulness, using frameworks like Han’s taxonomy or the Cynefin framework, regulating emotions through stress management, and building strong clinician-patient relationships through effective communication and empathy [28, 29, 30]. Peer-to-peer debriefing after communication skills sessions can help analyze and apply these strategies.

Given these theories, we advocate for the early exposure of medical students to the concepts surrounding medical uncertainty, ideally prior to entry into the clinical setting. This foundational understanding can better prepare them for the challenges they will face throughout their medical careers.

• Tip 2: Curriculum design

Papanagnou et al. developed an uncertainty-focused curriculum that significantly improved students’ ability to handle uncertainty, communicate with patients, and maintain well-being [23]. The curriculum included team debriefs, role-plays, case-based learning, and narrative sharing. Students valued storytelling, faculty role-modeling, simulations, and debriefings. The authors suggested that integrating these educational formats into formal curricula could help students develop skills for managing uncertainty.

Papanagnou et al. advocate for integrating Health Systems Science (HSS) into medical education, with uncertainty as a core theme [31]. HSS, the third pillar of medical education alongside basic and clinical sciences, provides a framework for understanding healthcare delivery complexities. It equips learners with competencies in value-based care, population health, interprofessional collaboration, systems thinking, and health system improvement. HSS prepares future healthcare professionals to navigate the complex, adaptive challenges of modern healthcare systems.

### Action

***Incorporate*** into the curriculum specific learning activities such as clinical debriefs, interprofessional role plays, simulations, communications skills training, instructor emotional vulnerability, storytelling, and peer-to- peer conversations may have the most impact in developing uncertainty tolerance in the trainee. Many of these key techniques identified as most helpful for students by students themselves in the action component of tips [23].

### Incorporate complexity frameworks

Use tools like the Cynefin framework to help students categorize and respond to uncertainty, distinguishing between simple, complicated, complex, and chaotic contexts in clinical practice [27].

### Foster cognitive flexibility

Introduce systems thinking and complexity science to enhance students’ ability to adapt and innovate in uncertain situations.

### Leverage humanities and liberal arts

Use patient narratives and philosophical discussions to encourage lateral thinking and empathy.

### Train communication skills

Embed deliberate practice of communicating diagnostic uncertainty into curricula through role-play, OSCEs, and real-world simulations.

### Integrate HSS early and longitudinally

Equip students with problem-solving, team collaboration, and leadership skills, using HSS to scaffold their ability to address uncertainty throughout their training.

### Promote shared decision-making

Teach students how to involve patients in navigating diagnostic and treatment uncertainties to improve patient-centered care.

### Adapt lessons from the COVID-19 pandemic

Build on the adaptability and innovative approaches observed during the pandemic to create resilient learners prepared for future challenges [31].

• Tip 3: Normalizing the discomfort uncertainty has on people

Strategies which promote normalization of the discomfort uncertainty has on students should be developed. Destigmatizing and normalizing the discomfort felt with uncertainty may be accomplished by fostering humility, flexibility and courage in managing uncertainty [24].

### Fostering humility

This involves communicating medical uncertainty and developing epistemic maturity. Students must learn that definitive answers aren’t always available, and an iterative, patient-inclusive approach is often necessary [24]. Michalec et al. found that humility aids in managing uncertainty by promoting trust in abilities and enabling adaptability [32]. They suggest formal humility training may better prepare clinicians for uncertainty and recommend further research on humility’s role in various clinical situations and types of uncertainty.

### Fostering flexibility

This consists of promoting a multifaceted approach that encompasses curricular integration, experiential learning, mentorship, and emotional training. By implementing these strategies, medical schools can better prepare students to navigate the complexities and uncertainties they will face in their future careers, ultimately leading to more effective uncertainty management and hopefully better patient care [24, 33].

### Fostering courage

This consists of palliating uncertainty and sharing uncertainty with a focus on the relational and emotional support to cope with uncertainty [34]. Fostering courage in medical students to manage uncertainty involves creating a supportive learning environment, engaging in simulation-based training, encouraging reflective practices and an open dialogue about fear, fostering a growth mindset and promoting mentorship. By implementing these strategies, educators can help students develop the courage necessary to face the inherent uncertainties of medical practice confidently and effectively.

### Action

Fostering attributes of humility, flexibility and courage may prove transformative for the medical student. Actioning these attributes in action including vis-à-vis reflective exercises, structured debriefings, or mindfulness practices lead to the holistic education and development of the student as a whole person, not just in tolerating uncertainty.

• Tip 4: Portfolio development and reflective journaling

Guide medical students/learners in developing a portfolio of uncertainty cases. Meyer and Willis report reflexive journaling can help understand the nature and wide variety of uncertainty and how solutions can be found [35]. The nature of the uncertainty and the potential direction of uncertainty propagation (how one navigates through the process of managing uncertainty) can be mapped. The actual course of clinical outcome can be overlaid with the anticipated potential propagation and these can be resolved.

### Action

*Encourage* learners to maintain a journal documenting cases where they experienced uncertainty. Each entry should include a clinical vignette, the specific area of uncertainty, management options considered, the chosen option, the outcome, and reflections on how they managed the uncertainty and lessons learned. The reflection process can also be structured by guiding students to document key areas of uncertainty, decision pathways, emotional responses, and lessons learned. Furthermore, comparison of journals through different years of training will allow the trainee visualize their maturation and development in managing uncertainty and growing as a clinician (McMahon et al. 2022) [36]. Including faculty-guided discussions would also maximize learning in these sessions.

Clinicians say they spend a lot of time internally processing uncertainty before decision-making—so reflective journals could be a part of this, if trainees had a facilitated critical reasoning activity to complement it, like self-explanation [37]. The consultant can correct inaccuracies, promote reasoning and explain their own thinking in approaching the problem, to direct trainees away from common cognitive biases like heuristic ambiguity [38].

### Building resilience and empathy through self-regulation and reflection

This category includes strategies that foster resilience and empathy for healthcare professional trainees by emphasizing reflective and self-regulatory practices. The focus is on helping trainees process uncertainty emotionally and cognitively while showing empathy for themselves and their patients. These strategies normalize uncertainty and encourage collaboration and introspection.

• Tip 5: Employ “think aloud”

Implement mentorship programs where learners can shadow senior practitioners and observe how they handle uncertainty [39]. We propose “think aloud” by senior clinicians where they talk through their process of critical analysis and decision-making. This is a specific self-regulatory practice and refers back to externalizing the senior clinicians process in decision-making. Thinking aloud is a measure of the process of cognition [40]. The process allows the student to witness that clinical reasoning is an iterative process examining and re-examining the data until a final diagnosis is secured [41].

### Action

*Shadowing* more experienced colleagues. A buddy system for medical trainees [42, 43] on the wards or in the outpatient clinic where they are assigned to a junior doctor allows the trainee witness how pervasive uncertainty is within clinical practice in staff not too much older than themselves [42]. Shadowing more senior consultant colleagues reinforces that everyone lives with uncertainty. Useful tips and tricks employed by senior practitioners highlights how experience equips doctors with coping mechanisms for managing uncertainty [44]. Curb-siding colleagues, revisiting diagnoses, reviewing data a second time, organizing follow-up and leaving the door open are all strategies which equip senior practitioners manage their uncertainty. Senior clinicians “think aloud” internally. This is a specific self-regulatory practice and refers back to externalizing the senior clinicians process in decision-making. Such clinicians can demonstrate how they structure and model their “thinking aloud” process. Facilitate peer-to-peer exercises, where students practice verbalize their thought processes while solving cases in small group settings where feasible.

• Tip 6: Peer-to-peer challenges on uncertainty

Peer to peer feedback among medical students can be highly effective, promoting self-reflection and improving communication skills [45]. It encourages open discussions about clinical uncertainty, teaching students to seek consultation when needed and accept that not having all answers is normal. This approach provides relevant, actionable feedback and fosters a culture of collaboration in medical education [46].

### Action

*Facilitate* monthly meetings for small groups of medical students to discuss challenges encountered during clinical rotations. Each student can share specific areas of uncertainty and describe their efforts to resolve these issues, such as consulting peers, seeking advice from practitioners, or reviewing medical literature (Pubmed, Medline, ERIC etc.). Story-telling serves as an effective way for students to convey their experiences and challenges in managing uncertainty [23]. Specific prompts can trigger meaningful sharing of stories between small groups.

• Tip 7: Mindfulness training and empathy

Incorporate mindfulness training into the curriculum to help learners become comfortable with uncertainty [47]. This demonstrates empathy for the student or “self-empathy.” Teaching the medical students how to show empathy for the patient is a critical strategy to proving caregivers with comfort with doctors’ reassurance [48]. Patients who derive empathy from their reveal more about their symptoms and concerns even improving the accuracy of their diagnosis [49]. Despite this there is a reduction in empathy expressed by the students as they graduate in years through medical school education [50]. There is increasing evidence that educational initiatives can augment the affective emotional responses of such students [51]. Furthermore, the negative effects of uncertainty on students can also be reduced through self-compassion [52].

### Action

*Recognize* that uncertainty often generates anxiety and worry for the student. Mindfulness techniques and exercises can help medical students manage anxiety from clinical uncertainty. Short pre-interaction mindfulness sessions and breathing exercises can reduce stress. Empathy workshops using patient narratives and personal simulations help students develop emotional skills. Employing empathy in managing caregivers (parents of children) is an important skillset to mitigate against uncertainty and build CDR [21]. Self-compassion is an important self-protective mechanism for students against negative effects of uncertainty.

### Enhancing communication and relationship-building skills

Effective communication is crucial for building trust and providing reassurance to patients and caregivers (parents of children) [21]. These tips focus on equipping trainees with skills to actively listen, clearly explain findings, and establish trust with patients. They highlight relational techniques that help both patients and caregivers feel reassured in the face of medical uncertainty.

• Tip 8: Master interpersonal communication

Effective healthcare delivery and managing uncertainty centers on the mastery of interpersonal communication [53]. This fundamental skill set, which encompasses active listening [54, 55], close observation, and careful language choice, ensures that caregivers (of children) are well-informed about potential red flags [56] and reassuring findings, fostering a sense of understanding and trust [21]. Here, the focus is on equipping medical students with the necessary skills to handle this crucial aspect of healthcare delivery effectively.

Our approach is to integrate educational pedagogies that emphasize experiential learning, continuous feedback [57], and real-world application [58]. Rising et al. showed the use of an Uncertainty Communication Education Module, a simulation-based mastery learning curriculum designed to establish competency in communicating diagnostic uncertainty, increased competency in communicating this in the emergency room setting [59]. Khazen et al. showed that a diagnostic uncertainty communication tool was successfully designed and implemented during clinical encounters in the general practice arena [60]. These strategies go beyond theoretical learning, immersing trainees in the practice of communication, helping them understand its nuances, and continuously striving for improvement.

### Action

***Role-modeling of communication strategies*** can facilitate this goal. Communication workshops can teach medical students key skills: active listening, clear explanation of the problem, and empathetic patient interaction. Focusing on succinct questioning and giving patients space to speak builds trust and the patients feel “cared for” not just “processed.” Trainee reflection on whether they truly actively listen is a powerful component of this training.

• Tip 9: Walking through the findings for reassurance

Training medical students to “walk through the findings” is crucial for effective patient and caregiver (parents of children) communication [21]. This skill involves explaining clinical observations logically, addressing concerns, and providing reassurance where appropriate. By building confidence in the absence of red flags or outlining a clear plan when red flags are present, clinicians can foster trust and alleviate anxiety [61].

By exposing students to uncertainty, enhancing their critical thinking, encouraging decision-making under uncertainty, and fostering effective communication, educationalists can help students develop the resilience and confidence needed to manage the challenges of clinical practice [8]. This comprehensive approach not only prepares them for real-world challenges but also imbeds a mindset that values learning and adaptation in the face of uncertainty [62].

### Action

Use real patient cases recorded during clinical interactions to practice this skill. For example, focus on common presenting complaints such as murmurs, chest pain, or syncope. Highlight how to explain findings to the patient and caregivers, reassuring them when no red flags are present or detailing the next steps when further investigation is warranted.

## Example of a Clinical Scenario: Walking Through the Findings

A thriving 2-year-old boy is referred with a 2/6 vibratory systolic murmur at the left lower sternal edge. The caregiver is distressed, as a neighbor’s child suffered a tragic cardiac event. The goal is to provide a thorough assessment and reassurance.

### Walking Through the History

Begin by acknowledging the caregiver’s concerns. Explain that the child is healthy and thriving with no cardiac symptoms or significant family history of inherited or congenital conditions. Reassure them that the neighbor’s tragic case, while understandably concerning, is unrelated.

### Walking Through the Examination

#### Highlight the Absence of Red Flags

Normal vital signs, pulses, precordium, heart sounds, and lack of heaves, thrills, or organomegaly. Describe the murmur’s characteristics, emphasizing its benign nature (e.g., a Still’s murmur that diminishes with the Valsalva maneuver). Build reassurance through effective communication, active listening and demonstrating empathy.

#### Actively Listen to the Caregiver’s Worries and Show Empathy for Their Distress

Use clear, lay-friendly language to explain why the findings are reassuring. Safety-net by arranging follow-up visits and, if necessary, offer additional testing such as an electrocardiogram. Explain how the results (e.g., normal axis and voltage, absence of long QT or Brugada syndrome) further support the benign diagnosis.

#### Summarize the Discussion

Recap the findings: the child’s good health, the benign murmur, and the absence of red flags. Explain why innocent murmurs occur and emphasize the plan for follow-up. Ask caregivers if they feel reassured, addressing any lingering concerns with patience and clarity.

By combining clinical expertise with compassionate communication, students can build both caregiver confidence and trust in the doctor’s reassurance, fostering a strong patient-clinician relationship.

• Tip 10: Trust-building for reassurance

Integrate trust-building exercises into the curriculum to teach learners the critical role of trust in fostering strong relationships with patients and their caregivers (including caregivers of children). Trust enhances both clinician and patient comfort with uncertainty and builds confidence in the care process [21]. Chew et al. emphasize that trust improves outcomes, even in scenarios like remote blood pressure monitoring, by addressing concerns openly and collaboratively [63].

##### Action

Encourage trainees to document and reflect on instances where they actively build trust with patients and caregivers. This can include moments where they:

Honestly communicate what is known, unknown, or uncertain. Avoid overly authoritative approaches and instead prioritize transparency and shared decision-making. Emphasizing transparency strategies, such as honest upfront disclosure of the pervasiveness of uncertainty builds trust and patient/caregiver confidence. Reassure patients by providing clear explanations about their condition, the next steps, and the reasoning behind recommendations. This reflective practice, part of part-task training, helps learners recognize and refine their trust-building strategies. For example, acknowledging uncertainty while emphasizing a plan of action demonstrates both competence and care. McMahon et al. also stress that trust is integral to reassuring caregivers [21]. When discussing uncertain or complex situations, clinicians should emphasize their ongoing support and dedication to finding the best outcome. Building trust strengthens relationships and reassures caregivers that the clinician is committed to addressing their concerns thoroughly and empathetically. By practicing these techniques, trainees can build confidence in their ability to reassure and support patients and caregivers effectively.

##### Clinical skills for reassurance and decision-making

These tips emphasize the importance of core clinical skills like history taking and physical examination in navigating uncertainty. These skills help trainees identify red flags and provide reassurance by demonstrating expertise and thoroughness. They also build the patient’s confidence in their care.

• Tip 11: History taking from the patient

Use of history taking from real patients (or caregivers in case of paediatrics) puts the medical student directly into the role of the doctor. Trying to take a comprehensive history including all the relevant positive and negative findings is a critical skill for medical students to learn. As the student progresses through the history, the presence of diagnostic uncertainty should become apparent [46]. McMahon et al. [21] previously identified a number of skills, including history taking, which were used in combination by senior clinicians which they believe assists patients and caregivers through uncertainty.

##### Action

*Evaluate* how the medical student takes a history from the real patient or parent of a child (in paediatric cases) with a classic presentation e.g. an innocent murmur, chest pain or syncope. The interaction is video-taped and the medical student watches back the interaction with the real patient. How the student utilizes red and white flags in dealing with uncertainty in common clinical cases facilitates learning. Breakdown each component of the history (presenting complaint, relevant positive and negative findings, importance of family/social history, systems review) in a step-by-step fashion identifying areas of uncertainty. Probe deeper into ambiguous symptoms, and reflect on how their history-taking approach would improve clarity. Faculty guidelines for structured feedback further enhances its educational impact.

• Tip 12: Physical examination for comfort

The physical examination plays a pivotal role in fostering patient (caregiver) confidence in their clinician [64–66]. Verghese and Horowitz highlight the examination as a scientific process that blends clinical skill with patient connection [65]. Trainees should prepare their physical examination techniques while embracing its ritualistic value, ensuring patient comfort throughout the process. This includes actively listening to the patient’s story without interruptions, carrying the appropriate tools, and approaching the patient with respect and kindness—all key elements that enhance bedside care.

Meyer et al. emphasize that involving patients by explaining physical findings and diagnostic steps fosters comfort with clinical uncertainty [67]. McMahon et al. add that walking caregivers, particularly in paediatric settings, through findings builds trust and comfort in the doctor’s reassurance, strengthening the overall caregiving experience [21].

Medical students should be encouraged to recognize cues that help mitigate uncertainty, an invaluable skill for building confidence [68, 69].

##### Action

Emphasize the importance of verbal communication to the patient and caregiver during the examination. Teaching students how to explain findings in real time, differentiating between concerning and benign signs, and involving caregivers in the process would enhance the skill’s application. Debrief clinical encounters with a focus on physical findings and their relationship to uncertainty. This debriefing process can involve senior clinicians as well as peer discussions. Highlighting *red flags* that warrant further investigation and *white flags* that help build comfort with uncertainty can be especially beneficial for students [69]. These discussions provide critical insights into decision-making processes, reinforcing both competence and patient-centered care.

## Discussion

A fundamental role for doctors, across specialties and clinical contexts, is providing reassurance [70]. This reassurance takes many forms: calming patients’ anxieties, alleviating caregivers’ fears, guiding medical trainees through their uncertainties, and even supporting colleagues who grapple with challenging cases. Recent research has increasingly focused on the role of uncertainty in clinical decision-making [36]. While uncertainty is a common experience for medical practitioners, a growing body of literature provides practical strategies for managing it effectively [22, 71].

Two notable works—Scott et al. (2019) [71] and Stephens and Lazarus [9]—offer crucial insights into how trainees can develop confidence and adaptability in the face of uncertainty. They emphasize the need for structured pedagogical approaches to help trainees not only manage their own discomfort but also reassure patients and caregivers. Yap et al. also describe different moderators for uncertainty tolerance [72]. Building on these contributions, this report offers 12 actionable tips organized around four core themes:

- **A. Understanding and integrating uncertainty into medical education**
- **B. Building resilience and empathy through self-regulation and reflection**
- **C. Enhancing communication and relationship-building skills**
- **D. Clinical skills for reassurance and decision-making**

This framework can also be particularly relevant in paediatrics, where the dual need to address the concerns of both children and their caregivers makes reassurance an indispensable skill. Central to this discussion is the concept of comfort with a doctor’s reassurance—an essential pillar of clinical practice that supports both the medical and emotional needs of patients and families. Effective reassurance mitigates anxiety, builds trust, and fosters collaborative relationships, particularly in moments of uncertainty. Safety-netting is an important step to provide parents with reassurance in the face of clinical uncertainty [73].

Comfort with a doctor’s reassurance is a cornerstone of effective clinical practice, addressing not only the medical needs of patients but also their emotional and psychological well-being. This skill is particularly important in moments of uncertainty, where trust and clear communication play a pivotal role in alleviating anxiety and fostering collaborative relationships between clinicians, patients, and caregivers.

One essential aspect of this reassurance occurs during physical examinations (Tip 12). These encounters offer a tangible way for clinicians to demonstrate attentiveness and care. By clearly explaining findings, walking patients through each step of the process, and using calm, empathetic language, doctors can provide immediate reassurance that their concerns are being taken seriously. This approach not only strengthens trust but also helps mitigate anxiety. Training in this area should emphasize how to frame findings honestly yet reassuringly, balancing transparency with sensitivity.

Another critical dimension involves explaining clinical findings and decisions systematically (Tip 9). Discussing the absence of red flags, empathizing with patient and caregiver anxieties, and outlining next steps are all key to building confidence in the medical process. This transparency demystifies uncertainty, transforming it from a source of fear into an opportunity for shared understanding. Clinicians must develop the ability to acknowledge uncertainties openly while reinforcing their commitment to navigating them collaboratively with patients.

Underlying these practices is the broader concept of trust-building (Tip 10). Effective reassurance stems from a foundation of open and honest communication. Clinicians should be trained to articulate what is known, unknown, and uncertain in a way that is both transparent and empathetic. This approach not only strengthens the clinician-patient relationship but also helps caregivers and patients feel empowered to engage actively in their care, reducing the emotional burden of ambiguity.

**Fig. 1:**
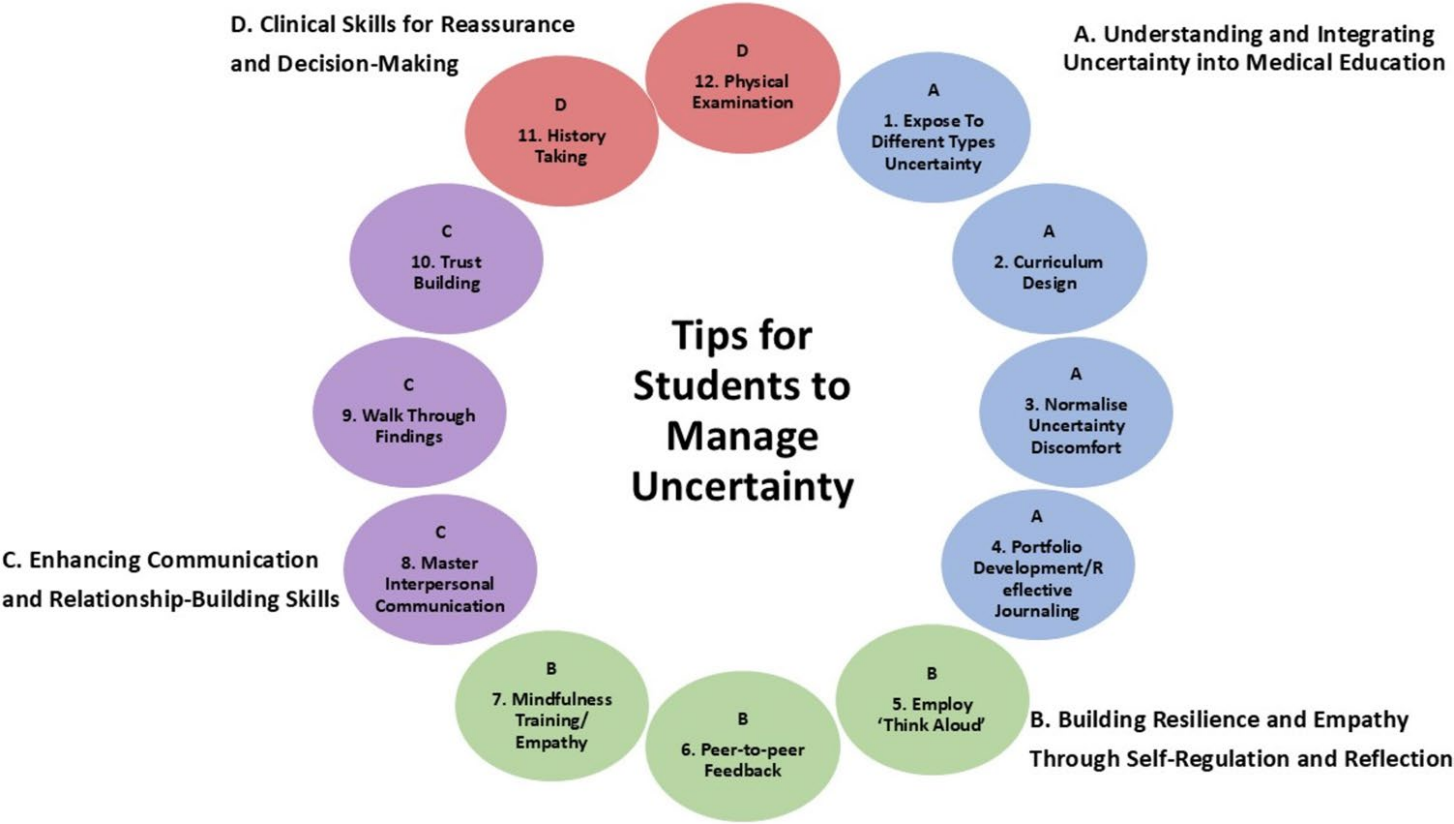
Schematic demonstrating the 4 domains and 12 tips for train medical students to manage their uncertainty and to provide reassurance to patients and their caregivers

Building on existing research, our paper advances the field by providing 12 practical, actionable tips organized into four key domains that address both the cognitive and emotional challenges of managing uncertainty (Fig. 1). Unlike prior work that often focuses on theory, our recommendations offer concrete strategies for medical students to develop empathy, resilience, and communication skills—especially important in paediatrics, where clinicians must support both patients and their caregivers. This structured approach bridges the gap between conceptual frameworks and real-world application, helping trainees navigate uncertainty with greater confidence and compassion..

### Closing the Loop by Linking Uncertainty and CDR Through Empathy and Caregiving

Paediatrics is a unique specialty in that there are effectively two sets of patients: the child and also their caregivers. Uncertainty is a challenging concept given how emotionally invested caregivers are in the well-being of their child [74]. Important strategies to provide CDR have been reported previously by our group [21]. Training healthcare professionals in the appropriate use of empathy/compassion and “providing care” for their patients helps mitigate against parental anxiety and promotes their CDR [75]. Coaching medical trainees in such techniques can mitigate uncertainty in a VUCA world [76].

### Theoretical Foundation to These Pedagogical Strategies

In this context, the Four-Component Instructional Design (4 C/ID) model offers a robust framework for fostering both the transfer and teaching of complex skills in medical education [79, 80]. The 4 C/ID model structures instructional design around real-life, whole-task learning, making it particularly effective for addressing the challenges of medical uncertainty and supporting holistic, dynamic learning experiences. While originally developed to teach domain-specific skills, such as clinical reasoning, the model is equally applicable to the development of broader, domain-general competencies, including communication and critical thinking [81].

Although the explicit focus of 4 C/ID is not on emotions or empathy, there is increasing recognition of their importance in complex learning environments. Instructional designers using the 4 C/ID approach are encouraged to integrate emotional considerations into learning tasks, supportive information, and coaching strategies to enrich the educational experience (4C/ID+E). For example, emphasizing authentic, progressively challenging clinical scenarios helps learners develop not only diagnostic skills but also the ability to empathize with patients and manage emotional responses to uncertainty [77, 78].

Resources such as simulations, mindfulness exercises, and structured debriefings can provide both cognitive and emotional support, helping learners to strengthen critical thinking while building emotional resilience. The model also underscores the value of expert mentorship and role modeling, where experienced clinicians demonstrate effective decision-making and uncertainty management, guiding learners through feedback, reflection, and direct coaching. This integrated approach cultivates cognitive flexibility, emotional resilience, and relational skills, all of which are essential for navigating the complexities of medical practice [77, 78].

## Conclusion

Uncertainty in medical decision-making is pervasive and challenging for the healthcare professional trainee. However, there are several concrete steps, as provided in this paper, which can build comfort with uncertainty for the trainee and reinforce the potential positive attributes of uncertainty in the holistic care of patients. Teaching healthcare professional learners to engage empathy and compassion in the care for their patients and indeed caregivers of patients may prove the most valuable tip of all.
